# Genomic characteristics and comparative genomics analysis of *Salmonella enterica* subsp. *enterica* serovar Thompson isolated from an outbreak in South Korea

**DOI:** 10.1038/s41598-022-22168-2

**Published:** 2022-11-29

**Authors:** Woojung Lee, Eiseul Kim, Hyunwoo Zin, Soohyun Sung, Jungha Woo, Min Jung Lee, Seung-Min Yang, Seung Hwan Kim, Soon Han Kim, Hae-Yeong Kim

**Affiliations:** 1 Division of Food Microbiology, National Institute of Food and Drug Safety Evaluation, Ministry of Food and Drug Safety, Cheongju, 28159 Korea; 2 Institute of Life Sciences & Resources and Department of Food Science and Biotechnology, Kyung Hee University, Yongin, 17104 Korea

**Keywords:** Biological techniques, Microbiology

## Abstract

*Salmonella* infections represent an important public health problem. In 2018, a multistate outbreak of *S. enterica* subsp. *enterica* serovar Thompson infection associated with contaminated chocolate cakes in schools was reported in South Korea. In this study, we sequenced the 37 *S.* Thompson strains isolated from chocolate cakes, egg whites, preserves, and cookware associated with the outbreak. In addition, we analyze the genomic sequences of 61 *S*. Thompson strains (37 chocolate cake-related outbreak strains, 4 strains isolated from outbreaks in South Korea and 20 strains available in the National Center for Biotechnology Information) to assess the genomic characteristics of outbreak-related strains by comparative genomics and phylogenetic analysis. The results showed that identically classified clusters divided strains into two clusters, sub-clusters A & I (with strains from 2018 in South Korea) and sub-clusters B & II (with strains from 2014 to 2015 in South Korea). *S.* Thompson isolated from South Korea were accurately distinguished from publicly-available strains. Unlike other *S*. Thompson genomes, those of chocolate cake outbreak-related strains had three *Salmonella* phages (SEN8, vB SosS Oslo, and SI7) integrated into their chromosome. Comparative genomics revealed several genes responsible for the specific genomic features of chocolate cake outbreak-related strains and three bacteriophages that may contribute to the pathogenicity of other *S*. Thompson strains.

## Introduction

*Salmonella enterica* is one of the most common foodborne pathogens, and it is responsible for > 99% of foodborne outbreaks caused by *Salmonella* species^1^. Over 2600 serovars of *S. enterica* have been identified, including Typhimurium, Enteritidis, Typhi, Paratyphi, and Bareilly^2^. *S. enterica* is the main foodborne pathogen that causes salmonellosis, which is characterized by diarrhea, vomiting, and high fever due to the consumption of contaminated foods. This species represents the second-most common and confirmed etiological agent, with 896 outbreaks and 23,662 hospitalizations reported between 2009 and 2015 in the United States^3^ and 6340 hospitalizations from 2014 to 2018 in South Korea, indicating that salmonellosis is one of the most common foodborne illnesses^4^.

*S. enterica* subsp. *enterica* serovar Thompson was first identified by Scott in 1926, and it belongs to the C1 serogroup^5^. *S.* Thompson has been identified as the cause of foodborne outbreaks associated with cilantro, arugula, chicken, beef, egg, bread, and smoked salmon consumption^6–8^. In addition, *S*. Thompson, *S.* Livingstone, *S.* Bareilly, and *S.* Montevideo were isolated at increasing frequencies and implicated in several foodborne disease outbreaks in 2014^9^. In September 2018, a large outbreak of *S.* Thompson infection associated with chocolate cake consumption was reported in South Korea. On September 6, the Ministry of Food and Drug Safety and the Korea Centers for Disease Control and Prevention (KCDC) announced that 13 schools in the country had reported outbreaks of gastroenteritis until September 5, and the same chocolate cakes provided to those schools were suspected as the source of infection. Subsequently, the distribution and sales of these chocolate cakes were suspended. Thereafter, the Ministry of Food and Drug Safety and the KCDC reported outbreaks of gastroenteritis in 57 mass meal services that distributed the chocolate cake in 12 provinces, including Seoul, until September 10, resulting in 2207 cases^10^. The genomic characteristics of *S.* Thompson need to be elucidated to further understand and control this newly emerging foodborne pathogen.

Advances in whole-genome sequencing (WGS) technology have made it an economically viable alternative to conventional typing methods for outbreak investigations and public health surveillance^11^. Comparative genomics using WGS data provide insights into the genomic characteristics of pathogenic bacteria, including candidate drug compounds, potential virulence determinants, mechanisms of pathogenicity, and their evolution in pathogens. In addition, several approaches are used to analyze WGS data for epidemiological and infection control purposes. A popular approach is to construct phylogenies based on single nucleotide polymorphism (SNP) variant calling, which identifies single nucleotide differences between isolates using a single reference genome^12,13^. An alternative approach is a gene-by-gene typing method, indexing core or accessory gene variation due to mutations or recombination events^14,15^. Consequently, this technology has been instrumental in improving diagnostics and public health microbiology^16^. Currently, WGS-based analysis is widely used to investigate outbreaks of pathogenic bacteria, such as *Bacillus cereus*, *Escherichia coli*, *Vibrio parahaemolyticus*, and *Salmonella* species^17,18^.

Major differences are found between and within species in their ability to cause infections. Horizontal transfer and acquisition of virulence factors are major driving forces in the emergence and evolution of pathogenic isolates. Several mobile genetic elements, such as insertion sequences, plasmids, bacteriophages, and pathogenicity islands, are implicated in the horizontal transfer of virulence genes; bacteriophages represent one of the most important factors^19^.

Bacteriophages are the most abundant biological entity on Earth and have been estimated to kill 20–25% of microbes daily^20,21^. Moreover, phages represent key contributors to bacterial ecology and evolution via obligate parasitism via either lytic or temperate life cycles, resulting in the direct or delayed lysis of bacterial hosts, respectively^22^. Phage–host interactions contribute considerably to genetic flux via horizontal gene transfer, which is responsible for the dissemination and acquisition of important bacterial phenotypes, such as enhanced colonization of the human gut epithelium, antimicrobial resistance, and toxin production^21,23^.

In this study, we aimed to characterize *S*. Thompson isolated from a chocolate cake-related outbreak in South Korea by WGS, utilizing various bioinformatics tools for outbreak analysis. The phylogenetic analysis using whole-genome multilocus sequence typing (wgMLST) and whole-genome SNP (wgSNP) analyses would provide their evolutionary relationships, and their typical phylogenetic patterns in South Korea would be determined. In addition, comparative genomic analysis revealed several genes responsible for the specific genomic features of chocolate cake outbreak-related strains and identified three bacteriophages. Consequently, this study would be useful to extend our understanding on the evolutionary relationship and pathogenesis of *S.* Thompson strains isolated from the chocolate cake-related outbreak in South Korea, and it would provide basic information on the control and regulation of this pathogenic *S*. Thompson for food safety.

## Results

### Genomic features

The representative genomes of *Salmonella* strains MFDS1011643 (chocolate cake), MFDS1011657 (egg white), MFDS1011659 (preserved foods), and MFDS1011716 (cookware) isolated from the food poisoning outbreak associated with contaminated chocolate cakes were sequenced; all strains had one chromosome and one plasmid in common (*Salmonella enterica* subsp. *enterica* strain YU39 plasmid pYU39_89 (accession number CP011430.1)) (Table 1). In silico serotyping predicted an antigenic profile of 7:k:1,5 *S.* Thompson (O antigen: 7, H antigen phase 1: k, H antigen phase 2: 1,5) based on the Kauffmann-White scheme. Furthermore, the sequence type (ST) was identified by using the *Salmonella* species multilocus sequence typing (MLST) database. *S*. Thompson strains MFDS1011643, 1011657, 1011659, and 1011716 were typed as ST26, which is associated with strains isolated from the chocolate cake-related outbreak in 2018.

**Table 1:** Genomic properties of *Salmonella enterica* serovar Thompson isolates from the chocolate cake-related outbreak in South Korea, 2018.

Genome features	MFDS1011643		MFDS1011657		MFDS1011659		MFDS1011716	
(Isolation source)	(Cake)		(Egg white)		(Preserved foods)		(Cookware)	
	Chromosome	Plasmid	Chromosome	Plasmid	Chromosome	Plasmid	Chromosome	Plasmid
Size (bp)	4,822,132	91,380	4,822,555	92,361	4,822,450	92,361	4,822,696	92,357
G + C content (%)	52.23%	47.22%	52.23%	47.29%	52.23%	47.29%	52.23%	47.29%
CDSs	4780	118	4770	118	4774	118	4776	118
rRNA (23S, 16S, 5S)	22 (8, 7, 7)	–	22 (8, 7, 7)	–	22 (8, 7, 7)	–	22 (8, 7, 7)	–
tRNA	83	4	83	4	83	4	83	4
GenBank accession no	JAKVTZ000000000		CP092627	CP092628	CP092510	CP092511	CP092690	CP092691

*CDS* Coding sequence, *rRNA* Ribosomal ribonucleic acid, *tRNA* Transfer ribonucleic acid.

MFDS1011643 contained a 4,822,132-bp chromosome and a 91,380-bp plasmid that showed G + C contents of 52.2 and 47.2%, respectively. Gene prediction of this genome revealed 4898 protein-coding sequences, as well as 87 tRNA (transfer ribonucleic acid) and 22 rRNA (ribosomal ribonucleic acid) genes. Similar results were observed for strains MFDS1011657, MFDS1011659, and MFDS1011716, which contained chromosomes sized 4,822,555, 4,822,450, and 4,822,696 bp and plasmids sized 92,361, 92,361, and 92,357 bp, respectively; the chromosomes and plasmids of all of these genomes showed G + C contents of 52.2 and 47.3%, respectively. The chromosomes and plasmids of MFDS1011657, MFDS1011659, and MFDS1011716 contained 4888, 4892, and 4894 protein-coding sequences, respectively, and they identically harbored 87 tRNA and 22 rRNA genes.

### Phylogenetic analysis

To investigate the genetic diversity of chocolate cake-related outbreak strains, wgMLST and wgSNP were used for the phylogenetic analysis of *S*. Thompson strains. The dataset is composed of 61 *S*. Thompson strains genomes: (1) chocolate cake-related outbreak strains (N = 37); (2) *S*. Thompson strains isolated from outbreaks in South Korea (N = 4); (3) *S*. Thompson strains, which are available in the NCBI (accessed on April 12, 2022) (N = 20).

**Figure 1:**
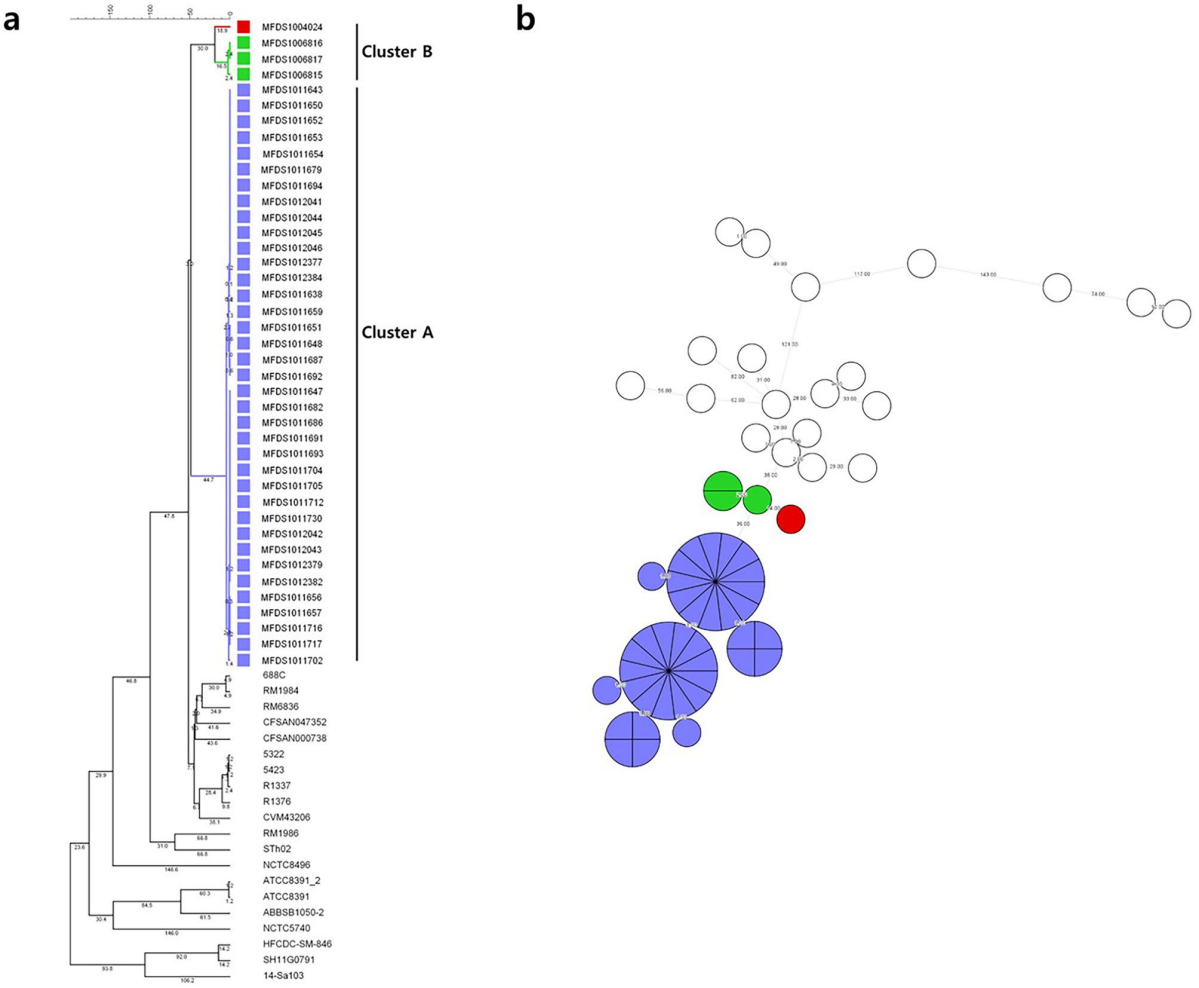
Whole-genome multilocus sequence typing (wgMLST) analysis of 61 *Salmonella enterica* serovar Thompson genomes. (**a**) Branches corresponding to outbreak isolates in 2014, 2015, and 2018 are shown in red, green, and purple, respectively, using an unweighted pair group method with an arithmetic-means-based tree. White squares represent strains obtained from the NCBI. (**b**) Circles corresponding to outbreak isolates in 2014, 2015, and 2018 are shown in red, green, and purple, respectively, in a minimum spanning tree. White rings represent strains obtained from the NCBI. The numbers on branches indicate wgMLST distances between isolates. Figure was generated using BioNumerics v. 8.0 (https://www.applied-maths.com/).

**Figure 2:**
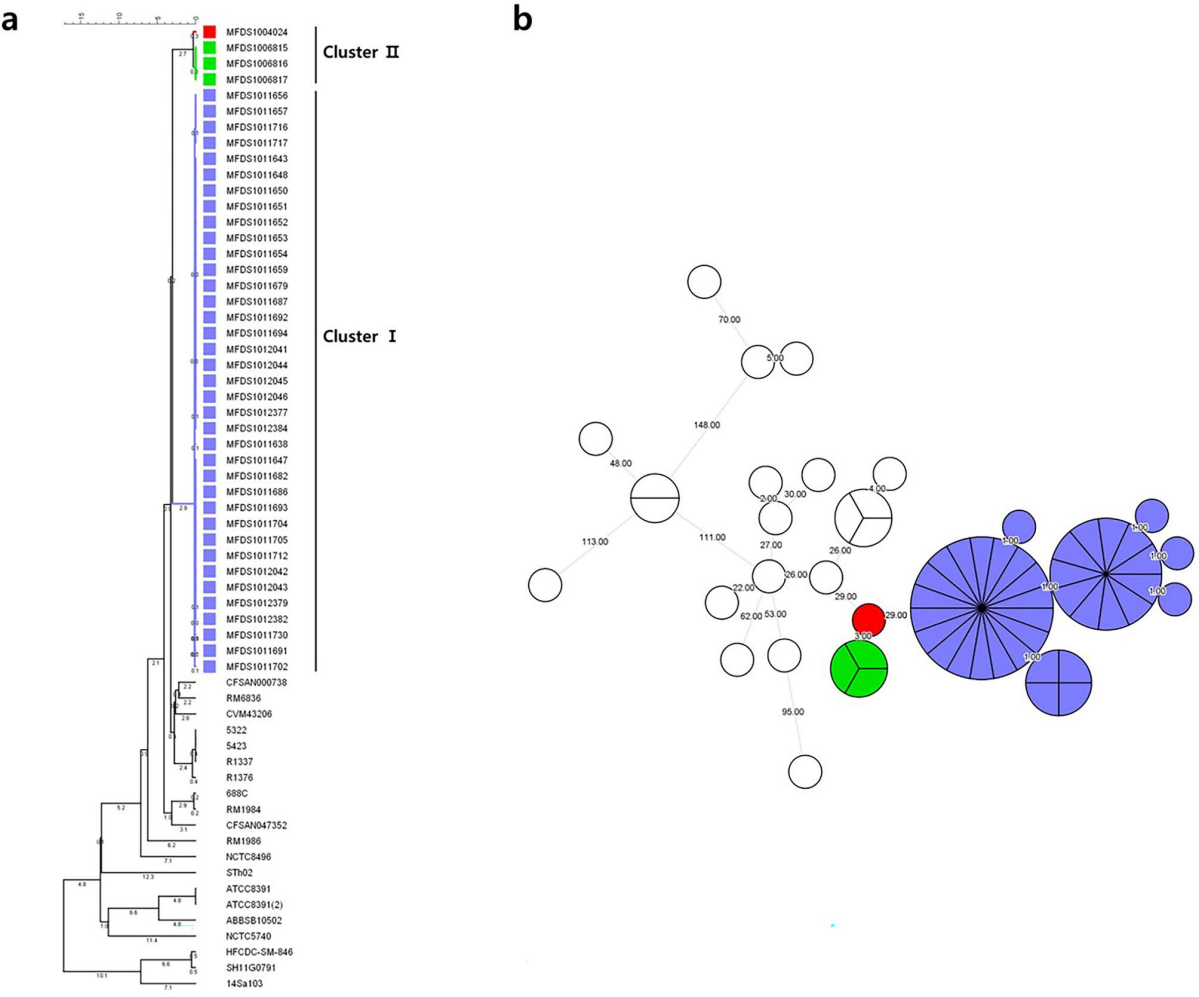
Whole-genome single nucleotide polymorphism (wgSNP) analysis of 61 *Salmonella enterica* serovar Thompson genomes. (**a**) Branches corresponding to outbreak isolates in 2014, 2015, and 2018 are shown in red, green, and purple, respectively, using an unweighted pair group method with an arithmetic-means-based tree. White squares represent strains obtained from the NCBI. (**b**) Circles corresponding to outbreak isolates in 2014, 2015, and 2018 are shown in red, green, and purple, respectively, in a minimum spanning tree. White rings represent strains obtained from the NCBI. The numbers on branches indicate wgSNP distances between isolates. Figure was generated using BioNumerics v. 8.0 (https://www.applied-maths.com/).

The phylogenetic tree was analyzed based on wgMLST and wgSNP. The wgMLST-based analysis showed that identically classified clusters divided strains into two clusters, cluster A (with strains from 2018 in South Korea) and cluster B (with strains from 2014–2015 in South Korea) (Fig. 1a). Cluster A isolates differed from each other by 0 to 5 alleles. Cluster A isolates differed from cluster B isolates by 40 to 54 alleles, and *S.* Thompson strains in the NCBI differed by 89 to 584 alleles (Fig. 1b). The results of the wgSNP-based analysis were the same as those of wgMLST. The wgSNP-based analysis showed that identically classified clusters divided strains into two clusters, cluster I (with strains from 2018 in South Korea) and cluster II (with strains from 2014 to 2015 in South Korea) (Fig. 2a). Cluster I isolates differed from each other by 0 to 3 SNPs. Cluster I isolates differed from cluster II isolates by 31 to 34 SNPs, and *S.* Thompson strains in the NCBI differed by 60 to 415 SNPs (Fig. 2b). The presence of 20 or fewer SNPs indicates that bacteria are genetically very similar and recently originated from the same source^24,25^. Therefore, the chocolate cake-related outbreak *S.* Thompson strains are distant from other isolates in South Korea. In addition, *S.* Thompson strains isolated in South Korea were accurately distinguished from *S.* Thompson strains reported in the NCBI.

### Comparative genomics

**Figure 3:**
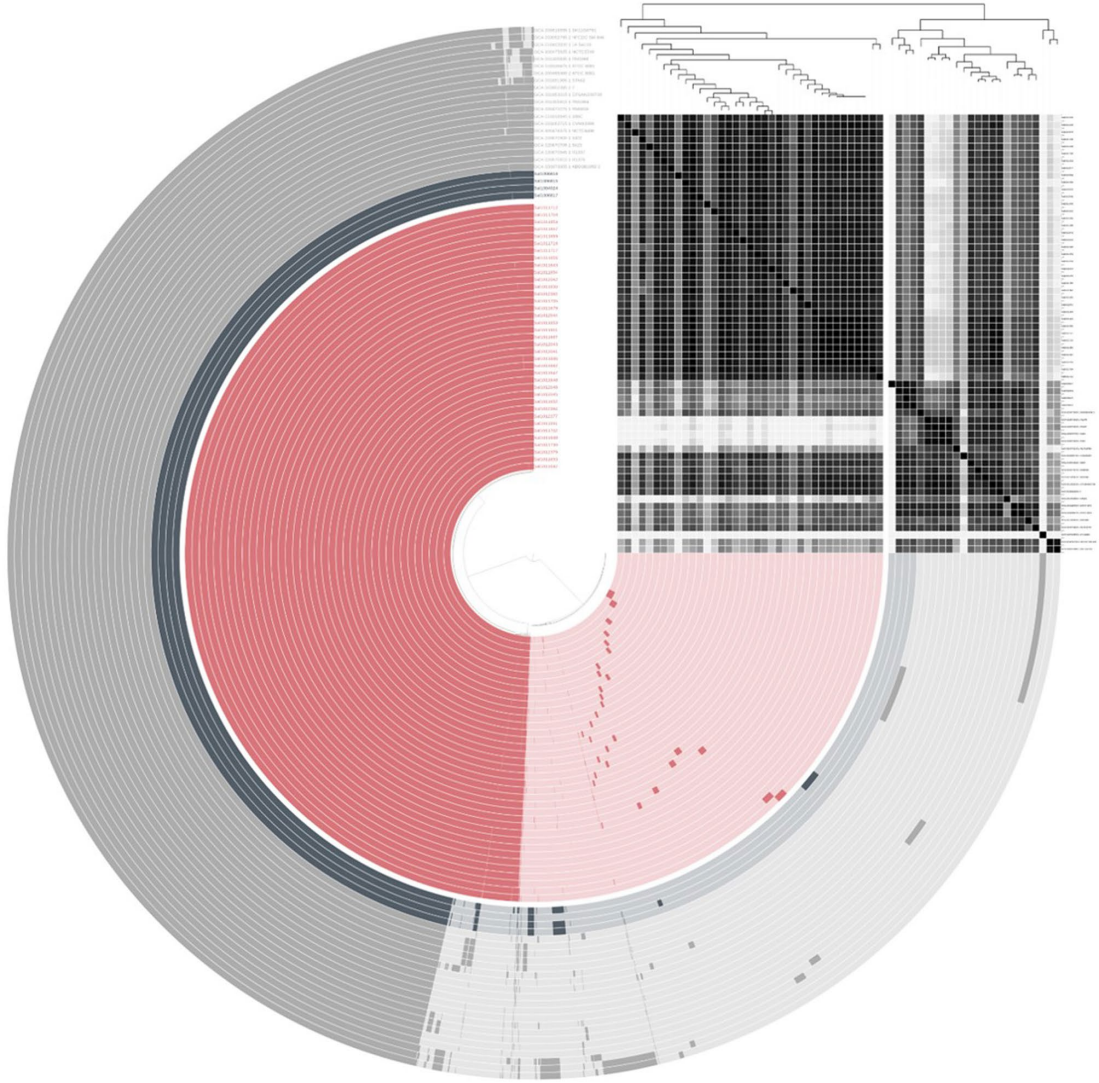
Pan-genome distribution of 61 *Salmonella enterica* serovar Thompson genomes. The color bar of pink, black, and gray represents chocolate cake *S.* Thompson outbreak-related genomes, other outbreaks in South Korea, and genomes obtained from the NCBI, respectively. In the layers, the dark and bright colors indicate the presence and absence of genes, respectively. Average nucleotide identity is represented as a heatmap showing high (black) and low (gray) similarity on the upper right region in the figure. Figure was generated using Anvi’o v. 6.0 (https://anvio.org/).

The serotypes of all genomes were identified as 7:k:1,5 (S. Thompson) by in silico serotyping. Also, all genomes were typed as ST26. All genomes were assigned to two distinct groups in the phylogenetic analysis based on pangenome. The first cluster contained 37 outbreak-related strains and the second cluster contained 24 other strains (Fig. 3). Pan-genome analysis showed that 61 whole-genome sequences of *S*. Thompson strains had a pan-genome comprising 7226 genes, which was segregated into a core genome of 3740 genes, an accessory genome of 1657 genes, and a unique genome of 1829 genes. Of the 1657 accessory genes, 121 were identified as unique genes in the 37 outbreak strains, including replication endonuclease, head protein, phage tail protein, and hypothetical proteins. Unique genes of outbreak strains were assigned nine COG categories: cell cycle control, cell division, chromosome partitioning (D); cell wall/membrane/envelope biogenesis (M); cell motility (N); signal transduction mechanisms (T); transcription (K); replication, recombination, and repair (L); nucleotide transport and metabolism (F); general function prediction only (R); and function unknown (S). Functional categories, such as general function prediction only (R, 20.94%), transcription (K, 15.71%), and replication, recombination, and repair (L, 15.71%), were the most enriched among the unique genes of the outbreak strains.

### In silico identification of virulence genes and antimicrobial resistance genes

Virulence gene mapping of 61 genomes was performed to determine the virulence repertoire of *S*. Thompson. In the 61 genomes, 162 virulence genes, including fimbrial adherence determinants, non-fimbrial adherence determinants, macrophage-inducible genes, magnesium-uptake genes, and regulation and secretion system genes, were identified. The detailed results of virulence gene mapping for the 61 *S*. Thompson genomes are shown in Supplementary Table 1. The virulence gene pattern of *S*. Thompson was similar for each strain, and a core set of virulence genes was conserved in all strains. Therefore, the genomes of *S*. Thompson isolated from the chocolate cake-related outbreak contained virulence factors similar to those present in other *S*. Thompson genomes.

All genomes including *S*. Thompson isolated from the chocolate cake-related outbreak carried SPI-1 and SPI-2 (Supplementary Table 1). Multiple virulence factors have been implicated in *Salmonella* pathogenesis. These factors include type 3 secretion systems (T3SSs) encoded in SPI-1, SPI-2, and other SPIs, as well as other factors, such as flagella, capsules, plasmids, and adhesion systems^26,27^. Among these factors, fimbriae play a critical role in virulence by facilitating the interaction of bacteria with host cells and other solid substrates^28^. Additionally, *S. enterica* produces two T3SSs encoded by SPI-1 and SPI-2^29^. The T3SS-1 cluster is responsible for the early phase invasion of intestinal epithelial cells and M cells in the gut lumen and the activation of proinflammatory responses. In contrast, T3SS-2 is associated with the late phase of infection, including intracellular survival and replication within host phagocytes^30^. Thus, *S*. Thompson strains isolated from the chocolate cakes harbored various virulence factors that may contribute to human pathogenicity.

The ResFinder database was used to forecast the antimicrobial resistance genes that 61 *S*. Thompson genomes contained. At least two or more antimicrobial resistance genes were present in every genome (Supplementary Table 2). All genomes including 37 chocolate cake-related outbreak strains had genes associated with aminoglycoside (amikacin and tobramycin). Among them, five genomes (14-Sa103, 688C, ABBSB1050-2, HFCDC-SM-846, and SH11G0791) possess multiple antimicrobial resistance genes, such as beta-lactam, tetracycline, and quinolone.

### Phage characterization

**Figure 4:**
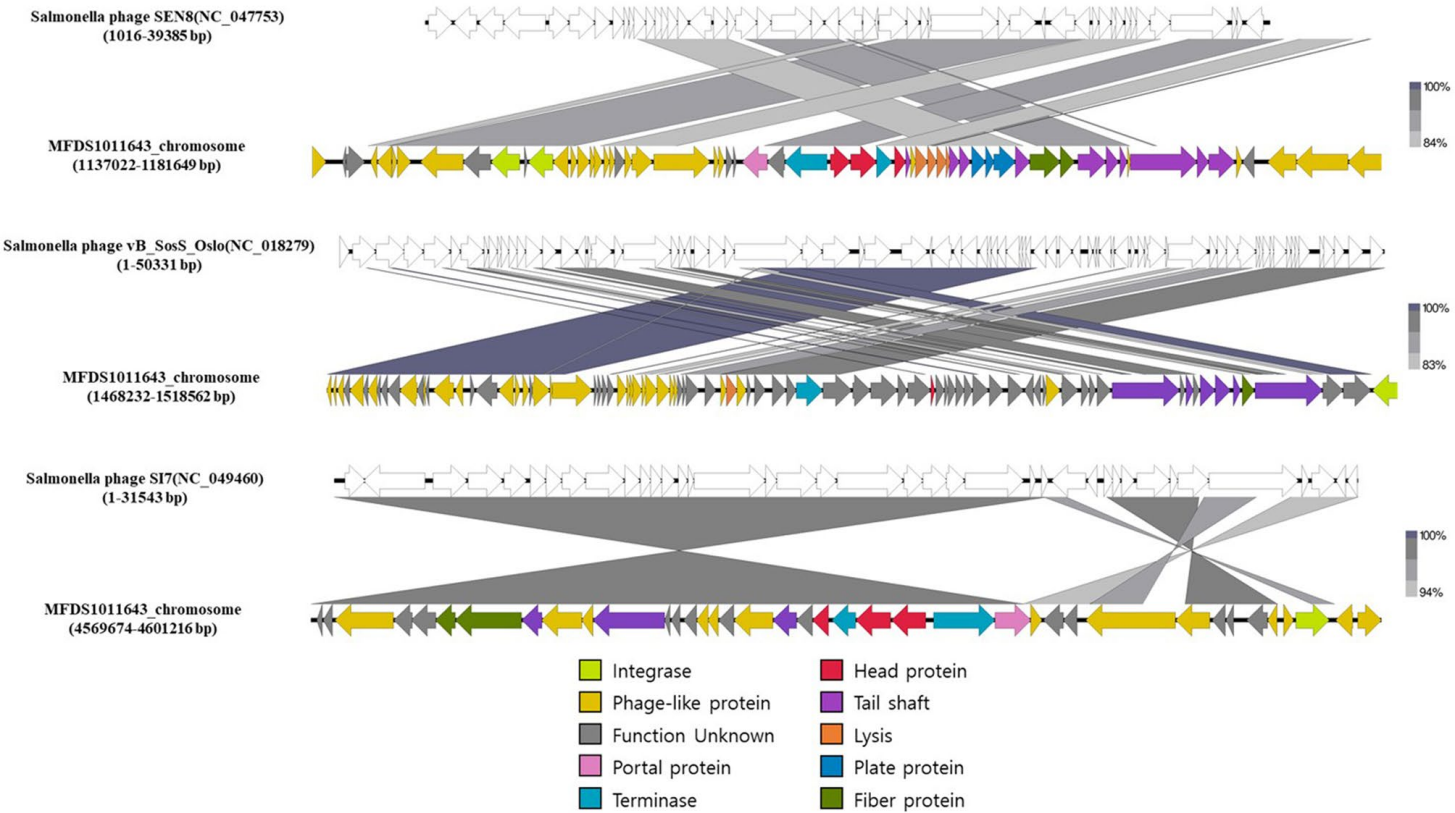
Genomic comparison between phages SEN8, vB_SosS_Oslo, and SI7 of MFDS1011643 strain, which was the main source of infection, and the most closely related phages (NC_047753, NC_018279, and NC_049460). Phage genomes are presented via linear visualization, with coding regions shown as arrows. Selected open reading frames are colored in relation to their functions**,** as indicated in the legend at the bottom. The percentage of sequence similarity is indicated by the intensity of the gray color. Figure was generated using Easyfig v. 2.2.5 (https://mjsull.github.io/Easyfig/).

Based on a comparison of genome sequences of 61 *S*. Thompson isolates, 37 *S*. Thompson isolates from the chocolate cake-related outbreak showed sequences of three unique bacteriophages in their chromosomes (Fig. 4). Based on the analysis of phages in 50 *S.* Thompson genomes, the 38.3-kb *Salmonella* phage SEN8 (accession number NC_047753), 50.3-kb *Salmonella* phage vB_SosS_Oslo (accession number NC_018279), and 31.5-kb *Salmonella* phage SI7 (NC_049460) were found only in the genomes of *S.* Thompson strains isolated from the outbreak. Therefore, while *Salmonella* pathogenicity islands (SPI) distribution was conserved among the evaluated *S*. Thompson genomes, the bacteriophage repertoire was diverse and contributed significantly to the genetic diversification of *S*. Thompson strains.

The purpose of this study is to predict the putative prophage sequence based on the genome sequence using in-silico phage search tool. Bacteriophage sequences were identified using PHASTER. PHASTER was used to predict putative prophage regions as “intact,” “questionable,” or “incomplete” based on the proportion of phage-related genes in the identified phage region. Prophages classified as “intact” by PHASTER and are the most likely to be complete and functional. In this study, the identified putative prophages were classified as “intact”. However, in order to confirm whether the identified prophage is induced or inactivated, excision induction experiments may need to be performed further.

## Discussion

*S*. Thompson often causes human diarrheal disease and has the ability to spread virulence and antibiotic resistance genes^31^. Therefore, in-depth studies for *S*. Thompson of genetic characteristics such as mobile gene elements and virulence factors are needed. Comparative genomics using WGS data provide insights into the genomic characteristics of pathogenic bacteria, including potential virulence determinants. In this study, we sequenced and analyzed the *S.* Thompson strains isolated from this chocolate cake-related outbreak to understand the pathogenic characteristics of *S*. Thompson strains.

The WGS-based analyses are able to construct the highly resolved phylogeny needed to support the findings of the outbreak investigation. The application of WGS allowed clustering analysis of *S*. Thompson in an outbreak and wgMLST, wgSNP, and phylogenetic tree analyses. The wgMLST and wgSNP analysis showed that *S*. Thompson clones isolated from egg white, cake, preserved foods, and cookware had the same clonal origin. This result represents likely cross-contamination events where egg whites had been contaminated with *S*. Thompson clones found in the cake, preserved foods, and cookware. Also, phylogeny analysis showed that *S.* Thompson strains isolated from South Korea were accurately distinguished from *S.* Thompson strains in the NCBI. Consequently, wgMLST and wgSNP analyses showed significant differences in *S*. Thompson strains isolated from a multistate chocolate cake-related outbreak in 2018, suggesting that human-infecting *Salmonella* strains can cause symptomatic salmonellosis.

*Salmonella* genomes were prophages are abundant (roughly 9000 phage types)^32^, and many prophages carry virulence genes that encode proteins that play major roles in bacterial pathogenesis. Unlike other *S*. Thompson genomes, those of chocolate cake outbreak-related strains had three *Salmonella* phages (SEN8, vB SosS Oslo, and SI7) integrated into their chromosome. The phage SEN8 encodes *fljA*, which plays crucial roles in bacterial motility, chemotactic behavior, and host cell invasion as a virulence determinant^33^. The phage SEN8 also encodes T1SS-associated proteins (LapB, LapC, and LapE), which function in relatively simple pathways that enable the secretion of a diverse range of virulence factors. The phage vB_SosS_Oslo encodes holin and endopeptidase Rz. Holin and endolysin are important for host cell lysis^34^. Holins create holes in the cytoplasmic membrane, which serve as transport channels for endolysins that digest the peptidoglycan layer. In addition, Rz/Rz1-like proteins often enhance endolysin activity as accessory proteins^35^. The phage SI7 encodes the copper-sensing two-component system response regulator CpxR, which is involved in the virulence of uropathogenic *E. coli*^36^, *S.* Typhimurium^37^, and *Vibrio cholerae*^38^. Additionally, several studies have verified the role of CpxR in the antibiotic resistance of pathogenic bacteria. In particular, the phage holin and endopeptidase Rz were present simultaneously in the strains isolated from the chocolate cake-related outbreak. Our results show that chocolate cake outbreak-related strains had three unique *Salmonella* phages (SEN8, vB SosS Oslo, SI7) integrated into their chromosomal sequences, which contributed considerably to the genetic diversification of *S*. Thompson strains.

In conclusion, we reported the genome sequences and functional genomic features of 37 *S*. Thompson strains that were isolated from the large-scale outbreak associated with contaminated chocolate cakes in South Korea in 2018. Clustering and phylogenetic analysis from wgMLST and wgSNP suggested that *S*. Thompson strains isolated from a multistate chocolate cake-related outbreak in 2018 had low genetic relevance to previously reported *S*. Thompson strains^24,25^. In addition, comparative genomic analysis provides comprehensive information on the disease potential of the outbreak strains. Comparative genomic analysis showed that the outbreak strains were similar to previously reported *S*. Thompson strains, except that the outbreak strains contained a few different genes related to phages. However, further studies are required to elucidate the association between unique phage-related genes and large-scale foodborne disease outbreaks. Along with the identified genomic features of outbreak strains, the accumulation of additional genome sequence information on diverse *S*. Thompson strains can serve as basic information for preventing foodborne disease outbreaks and assisting treatment in South Korea. Also, the information obtained by WGS can be further used to gain insight into antibiotic resistance, virulence genes, and molecular evaluation for pathogenesis.

## Methods

### Strain isolation and serotyping

Thirty-seven *S*. Thompson strains associated with the chocolate cake-related outbreak in 2018 were isolated from chocolate cakes, egg whites, preserves, and cookware. The strains isolated in this study are summarized in Table 2. The samples were plated on xylose lysine deoxycholate agar (Oxoid, Basingstoke, United Kingdom) and Rambach agar (CHROMagar, Paris, France) and incubated at 37 °C for 18–24 h. After isolation, the isolates were identified using the VITEK MS and VITEK 2 systems using a GN card (BioMerieux, Marcy-l'Étoile, France). *Salmonella* serotyping was performed according to the White-Kauffmann-Le Minor scheme using somatic (O) (Denka Seiken, Tokyo, Japan) and flagella (H) antisera (Becton & Dickinson, Sparks, MD, USA).

**Table 2:** Information on *Salmonella enterica* serovar Thompson outbreak-related isolates.

Strain name	Collection date	Geographic location	Isolation source
MFDS1011638	2018-09-08	Gwangju	Cake
MFDS1011643	2018-09-08	Seoul	Cake
MFDS1011647	2018-09-08	Seoul	Cake
MFDS1011648	2018-09-08	Seoul	Cake
MFDS1011650	2018-09-08	Seoul	Cake
MFDS1011651	2018-09-08	Seoul	Cake
MFDS1011652	2018-09-08	Seoul	Cake
MFDS1011653	2018-09-08	Seoul	Egg white
MFDS1011654	2018-09-08	Seoul	Egg white
MFDS1011656	2018-09-08	Seoul	Egg white
MFDS1011657	2018-09-08	Seoul	Egg white
MFDS1011659	2018-09-08	Busan	Preserved foods
MFDS1011679	2018-09-05	Daegu	Preserved foods
MFDS1011682	2018-09-08	Busan	Preserved foods
MFDS1011686	2018-09-08	Busan	Preserved foods
MFDS1011687	2018-09-08	Busan	Preserved foods
MFDS1011691	2018-09-08	Jeju-do	Preserved foods
MFDS1011692	2018-09-08	Chungcheongbuk-do	Preserved foods
MFDS1011693	2018-09-08	Chungcheongbuk-do	Preserved foods
MFDS1011694	2018-09-08	Gyeongsangbuk-do	Preserved foods
MFDS1011702	2018-09-08	Gyeongsangnam-do	Preserved foods
MFDS1011704	2018-09-08	Gyeongsangnam-do	Preserved foods
MFDS1011705	2018-09-08	Gyeongsangnam-do	Preserved foods
MFDS1011712	2018-09-08	Ulsan	Preserved foods
MFDS1011716	2018-09-08	Seoul	Whipper
MFDS1011717	2018-09-08	Seoul	Whipper
MFDS1011730	2018-09-08	Jeollabuk-do	Preserved foods
MFDS1012041	2018-09-05	Gyeongsangbuk-do	Cake
MFDS1012042	2018-09-06	Gyeongsangbuk-do	Cake
MFDS1012043	2018-09-06	Gyeongsangbuk-do	Cake
MFDS1012044	2018-09-06	Gyeongsangbuk-do	Cake
MFDS1012045	2018-09-06	Gyeongsangbuk-do	Cake
MFDS1012046	2018-09-06	Gyeongsangbuk-do	Cake
MFDS1012377	2018-09-07	Gyeongsangnam-do	Cake
MFDS1012379	2018-09-07	Gyeongsangnam-do	Cake
MFDS1012382	2018-09-06	Gyeongsangnam-do	Cake
MFDS1012384	2018-09-06	Gyeongsangnam-do	Cake

### Genome sequencing, assembly, and annotation

Genomic DNA (deoxyribonucleic acid) was extracted using a MagListo™ 5 M Genomic DNA Extraction Kit for cells and tissues (Bioneer, Daejeon, Korea), according to the manufacturer’s protocol. The integrity and concentration of DNA were determined via standard agarose gel electrophoresis and a Qubit™ 3.0 Fluorometer (Life Technologies, Carlsbad, CA, USA), respectively. A DNA library was prepared using the Nextera DNA Flex Library Prep Kit (Illumina, San Diego, CA, USA). Sequencing was performed using a MiSeq sequencing system (Illumina) and a MiSeq Reagent Kit v3 (600-cycle). Raw read data were first demultiplexed and quality-trimmed to remove low quality reads and base calls in CLC Genomics Workbench using a modified Mott trimming algorithm and a parameter value limit of 0.05; ambiguous nucleotides were trimmed using a maximum number of ambiguities of two. The contigs (FASTA sequence files) were assembled de novo using CLC Workbench version 12.0 (Qiagen, Hilden, Germany) with automatic word size 20 and bubble size 50, and a minimum contig length of 200 bp. Paired read distances were automatically detected and contigs were scaffolded where possible. Following assembly, the reads were mapped back to the contigs using a mismatch cost = 2, insertion cost = 3, deletion cost = 3, length fraction = 0.5, and similarity fraction = 0.8; contigs were updated and gaps were filled. To obtain high-quality data to determine a complete genome sequence, hybrid genome assembly was performed using additional long-read sequence data obtained using PacBio Sequel (Pacific Bioscience, Menlo Park, CA, USA). Hybrid assembly for raw FASTQ PacBio sequel long-read sequence data and Illumina MiSeq short-read FASTQ sequence data were performed using Unicycler (v0.4.9, https://github.com/rrwick/Unicycler; default options). The assembled genome was annotated using the Rapid Annotation using Subsystem Technology toolkit as implemented in the PATRIC annotation web service (v3.6.12).

### DNA sequence analysis and bioinformatics

A comparative genomic analysis was performed for the 37 *S*. Thompson strains associated with the chocolate cake-related outbreak in 2018 and 24 other *S*. Thompson strains. The sequences of 4 strains isolated from other outbreaks^39^ in South Korea and 20 other *S*. Thompson strains are available in the National Center for Biotechnology Information (NCBI, accessed on August 06, 2021). The genomes analyzed in this study are summarized in Supplementary Table 3. The pan-genome was analyzed using the bacterial pan-genome analysis (BPGA) tool (v. 1.3; default parameters). The assignment of cluster of orthologous groups (COG) functional categories for genes derived from the pan-genome were performed using USEARCH in the BPGA tool, against the COG database. Virulence factors and antimicrobial resistance genes were identified using the virulence factor database and ResFinder v.4.1, respectively^40,41^. Plasmid features for complete genomes were confirmed by BLASTN search v. 2.13.0 (https://blast.ncbi.nlm.nih.gov/Blast.cgi). wgMLST was performed with BioNumerics 8.0 (Applied Maths, Sint-Martens-Latem, Belgium), where 15,874 loci for *S. enterica* were included for analysis. wgSNP analysis was performed with BioNumerics 8.0 (Applied Maths) using *S. enterica* LT2 as the reference genome, and “Strict SNP filtering” was applied. Clustering of the wgMLST and wgSNP results was performed by an unweighted pair group method with an arithmetic-means-based tree. A minimum spanning tree based on pairwise wgSNP differences was also constructed using BioNumerics. In addition, the web-based serotyping tool SeqSero 1.2^42^ was used to predict the antigenic profile of *Salmonella* strains.

### Prophage prediction and analysis

Bacteriophage sequences within the unique genome sequence were identified using PHAge Search Tool Enhanced Release (PHASTER)^43^. PHASTER was used to predict putative prophage regions as “intact,” “questionable,” or “incomplete” based on the proportion of phage-related genes in the identified phage region. Three intact prophage sequences were subjected to further analysis to confirm the prophage completeness. The basic local alignment search tool was used to align the three intact prophage sequences to those of *Salmonella* phages SEN8 (NC_047753), vB SosS Oslo (NC_018729), and SI7 (NC_049460) obtained from the NCBI database. Easyfig 2.2.5 was used to analyze the homologous regions between phage sequences^44^.

## Accession codes

The complete genome data of *Salmonella enterica* subsp. *enterica* serovar Thompson strains MFDS1011643, MFDS1011657, MFDS1011659, and MFDS1011716 have been deposited in GenBank with Accession No. JAKVTZ000000000, CP092627/CP092628, CP092510/CP092511, and CP092690/CP092691, respectively. The draft genome data of 33 *S*. Thompson strains associated with the chocolate cake-related outbreak in 2018 have been deposited in NCBI Sequence Read Archive (SRA) with Accession No. SRR18056016 ~ SRR18056048.

## Supplementary Information

Supplementary Information.

## Data Availability

Sequence data have been submitted to the NCBI archives (https://ncbi.nlm.nih.gov), including GenBank, Sequence Read Archive (SRA), under the accession numbers listed in Supplementary Table 3.
